# Severe long-term clinical sequelae among Sudan ebolavirus disease survivors 2 years post-infection

**DOI:** 10.21203/rs.3.rs-6325522/v1

**Published:** 2025-04-17

**Authors:** Haruna Muwonge, Carolyne Nasimiyu, Barnabas Bakamutumaho, Peter Elyanu, Moses L. Joloba, Silvia Situma, John Schieffelin, Bronwyn Gunn, Shuangyi Bai, Robert F. Breiman, Isaac Ssewanyana, Susan Nabadda, Julius Lutwama, Yonas Tegen, Allan Muruta, Bruce Kirenga, Charles Olaro, Jane Ruth Aceng, Henry Kyobe Bosa, M. Kariuki Njenga

**Affiliations:** Makerere University Medical School; Washington State University Global Health-Kenya; Uganda Virus Research Institute; Baylor-Uganda; Makerere University Medical School; Washington State University Global Health-Kenya; Tulane University; Washington State University Global Health-Kenya; Washington State University; Emory University; Uganda Central Public Health Laboratories; Uganda Central Public Health Laboratories; Uganda Virus Research Institute; Independent Global Health Consultant; Ministry of Health Uganda; Makerere University Medical School; Ministry of Health Uganda; Ministry of Health Uganda; Ministry of Health Uganda; Washington State University Global Health-Kenya

## Abstract

**Background:**

While long-term clinical sequelae following ebolavirus disease (EVD) due to Zaire ebolavirus (EBOV) strain has been characterized, this has not been explored for Sudan ebolavirus (SUDV) strain.

**Methods:**

We enrolled 87 SUDV survivors from the 2022–2023 outbreak in Uganda, alongside 176 age-, sex-, and location-matched controls. Clinical symptom data were collected at 3-, 9-, 12-, 15-, and 18-and 24-months post-infection. Serum, semen, and breast milk samples were collected and tested for viral RNA.

**Results:**

Of 86 SUDV survivors, 57.5% reported significantly higher frequencies of clinical symptoms involving musculoskeletal (45.0%, P < 0.001), central nervous system (36.3%, p < 0.001), ophthalmologic (20%, P < 0.001), and respiratory (10%, P < 0.001) systems than those observed among controls. The risk ratio of occurrence was highest for ophthalmologic (20% vs 3.4%, RR = 5.9; p < 0.001) and central nervous systems symptoms (36.3% vs 6.8%, RR = 5.3, p < 0.001), and lowest for reproductive system (13.8% vs 8.5%; RR = 1.6; p > 0.005). Importantly, 50% of SUDV survivors reported persistent multi-systemic symptoms, including low back pain, hand and feet numbness, confusion, and diarrhoea that resulted in inability to perform basic activities of living. Viral RNA was detected in semen for a median duration of 131 days (range: 111–210 days) and in breast milk for a median of 149 days (range: 111–199 days).

**Conclusions:**

This study demonstrates that SUDV survivors develop long-term clinical sequelae characterized by persistent multi-systemic clinical symptoms. Detection of viral RNA in semen and breastmilk for up to 7 months post-infection suggest prolonged persistence, with the possibility of latency and reactivation of the virus.

## Background

Ebola virus disease (EVD) is a severe, often fatal haemorrhagic fever in humans that is often caused by four ebolavirus strains; Zaire (EBOV), Sudan (SUDV), Bundibugyo (BDBV), and Tai Forest (TAFV) viruses (1, 2). While acute EVD has similar progression across the virus strains, with > 50% of cases developing life-threatening complications such as hypotension and acute multi-organ failure, the case fatality rate (CFR) ranges from 75–90% for EBOV, 55–65% for SUDV, to 25–33% for BDBV (2–4). Over the last 30 years, EBOV and SUDV have been the most prevalent strains, responsible for > 70% of EVD epidemics, all originating from Africa (1, 2). Recent studies, primarily in EBOV survivors, have described long-term EVD clinical sequelae characterized by sometimes severe and disabling musculoskeletal, neurological, psychological, ophthalmologic, and auditory symptoms that manifest from as early as 35 days after the acute EVD and can persist for years (5–7).

Following the 2014–2016 West African EBOV epidemic, studies identified a wide spectrum of long-term clinical sequelae, primarily with musculoskeletal and ophthalmologic symptoms (8–13). Sustained mental health impacts included post-traumatic stress disorder, depression, substance abuse, anxiety, psychosis, and suicidal ideation (14, 15). Ocular symptoms were associated with high viral load and severe acute EVD disease characterized by hemorrhagic manifestations (16). More recent studies demonstrated that host-specific anti-EBOV immune responses correlated with clinical sequelae in part by predisposing individuals to virus persistence in immune-privileged sites, eliciting inflammatory reactions that yielded long-term clinical manifestations (17). To investigate EVD clinical sequelae in survivors of SUDV strain, we enrolled 87 survivors from the 2022 outbreak in Uganda (18) shortly after discharge from Ebola treatment units (ETUs) and implemented long-term follow-up procedures. The outbreak, which started in September 2022 ended in January 2023, resulted in 142 confirmed SUDV cases, 55 deaths (CFR = 38.7%).

## Methods

### Study design and location

We conducted a matched cohort study starting from February 2023 following Sudan ebolavirus (SUDV) survivors from the September 2022 to January 2023 outbreak in Uganda. All 87 SUDV survivors were consented and enrolled together with 192 age-, sex- and location-matched community controls, and followed up at 3-, 9-, and 12–15- and 24 months post-infection. While a 2:1 control ratio was envisaged, we over-enrolled controls by 10% to account for possible SUDV seropositives, which were subsequently removed from the study after SUDV serologic testing.

**Figure 1:**
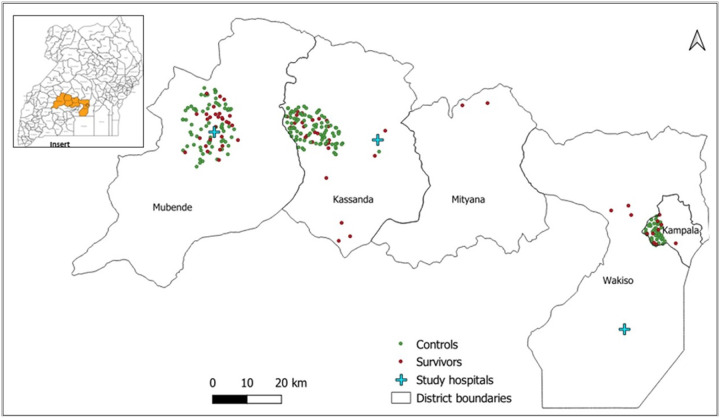
Spatial distribution of Sudan ebolavirus (SUDV) survivors and controls in Uganda. Map illustrating spatial distribution of SUDV survivors (red dots) and controls (green dots) across Mubende, Kassanda, Mityana, Wakiso, and Kampala districts. The study hospitals (blue crosses) include Mubende Regional Referral Hospital, Kikandwa Health Center III in Kassanda, and Entebbe Regional Referral Hospital in Wakiso. The insert map of Uganda showing location of affected districts (orange shading).

Participant follow-up was embedded within an ongoing clinical and psychosocial support program conducted by the MOH at three Uganda Ministry of Health (MOH) designated SUDV survivor clinics; Mubende Regional Referral Hospital, Kikandwa Health Centre III in Kassanda District, and Entebbe Regional Referral Hospital (Fig. 1). These health facilities are in the 3 districts that served as epicenters of the outbreak: Mubende, Kassanda, and Kampala districts.

### Participant enrolment

All 87-laboratory confirmed SUDV survivors were enrolled shortly after discharge from the Ebolavirus Treatment Units (ETUs). Location, age, and sex-matched community controls were recruited from five villages where 86% of the survivors lived, with the number of controls proportional to the survivor population in each village. To minimize the chance of recruiting controls, previously exposed to EVD, individuals living in the same households with survivors, having a history of EVD exposure or fever during the 2022 SUDV outbreak duration were excluded. The matching criteria for controls included location (same or nearby village), sex, and age (± 1 year for children < 5 years old, ± 2 years for participants 5–40 years old, and ± 5 years for those > 40 years). Controls were purposively identified through local council leadership and village health teams.

### Clinical data collection and sampling

Data were collected at enrolment and during follow-up visits using structured questionnaires, medical le abstraction, and physical examinations. For SUDV survivors, detailed information was gathered regarding their EVD illness, including clinical presentation, management, past medical history, known EVD exposure risk factors, persistent symptoms after discharge from the ETUs, and reproductive health history. Follow-up visits focused on updating the participants’ current health status, capturing any new symptoms, hospitalizations, exacerbations of existing conditions, and changes in sexual health. The questionnaires systematically collected symptom information, categorized by body system as summarized in Table 1.

Additional information regarding clinical signs, medical complications, laboratory test results, imaging findings, and treatments administered during ETU admission was abstracted from health records. Questionnaires were administered by trained study nurses, while additional targeted physical examinations were performed by a trained medical officer using the standard clinical methodology (19).

Venous blood was collected from each consenting participant (both survivors and controls), serum harvested and transported to Uganda Central Public Health Laboratory (CPHL) for storage. For women who were pregnant, placental blood, amniotic fluid, breast milk, and the baby’s tears and eye swabs and were collected at delivery. Eligible adult male participants provided semen while lactating females provided breast milk samples through self-expression.

### Collecting and testing semen and breast milk for SUDV

During each visit, eligible SUDV survivors were provided with specimen containers and instructed on a sterile collection of semen or milk samples by the trained MOH healthcare team conducting psychosocial follow-up. The specimens were triple packaged in accordance with international standards, refrigerated (at 5 to 8°C) and transported to the either the EVD mobile laboratory at Mubende Regional Referral Hospital, or the enhanced BSL-2 high containment laboratory at the Uganda Virus Research Institute (UVRI). At the mobile laboratory or UVRI, samples were placed into a negative pressure isolating glovebox where they were chemically inactivated using a mixture of virus lysis buffer and ethanol according to the manufacturer’s instructions (QIAmp viral RNA kit, Qiagen, Germany).

All PCR testing for SUDV was conducted at UVRI where viral RNA was extracted from 140 ul sample using QIAmp viral RNA kit (Qiagen, Germany) followed by amplifications cycling using using the BioPerfectus ebolavirus real time PCR kit (Jiangsu BioPerfectus Technologies, Taizhou, China) that detects both Ebolavirus Zaire (EBOV) and SUDV strains.

### Screening the control group for SUDV exposure

Serum samples collected from the 192 controls were assayed for IgG reactivity to recombinant SUDV glycoprotein (GP) and nucleoprotein (NP). Recombinant SUDV GP (IBT Bioservices, Cat# 0502 – 015) and NP (Sino Biologics, Cat# 40444-V07E1) were individually coupled to MagPlex (Luminex) magnetic beads with distinct fluorescent properties. Serum samples were diluted 100, 500, and 1000-fold in 1X PBS and incubated with the antigen-coated beads for 2 hours at room temperature at 900 RPM. Following incubation, the beads were washed 3X with 1X PBS + 0.1% Tween 20, and then incubated with 0.65μg/ml of PE-labelled secondary antibodies to detect IgG (total IgG, Southern Biotech, Cat# 2040–09) for 1 hour at room temperature at 900 RPM. Analysis was conducted on an IntelliFLEX SE-DR instrument (Luminex). A minimum of 30 beads per region were collected, and the median fluorescent intensity across the collected beads were recorded and reported.

A seropositivity threshold was determined based on the response of 24 seronegative controls comprised of 16 regional controls from Kenya and 8 US-based controls collected in 2020, which are presumably SUDV naïve. The threshold was calculated using the mean of responses + 5* the standard deviation across the samples at each serum dilution. Control samples that exceeded the threshold for SUDV GP across all three serum dilutions or across the 500- and 1000-fold dilutions (N = 16) were considered seropositive.

### Data analysis

Data cleaning and analysis were performed using R statistical software version 4.2.3. Descriptive analysis was conducted for categorical and numerical data and presented as frequencies, proportions, means and medians. Pearson’s chi-square test or Fisher’s exact test (where appropriate) were used to assess differences in symptom prevalence and other categorical variables between survivors and controls. Risk ratios (RRs) with 95% confidence intervals (CIs) were calculated to compare the likelihood of developing clinical sequelae among survivors relative to controls. For numerical variables, Student’s t-test or Mann-Whitney U test was used depending on data distribution, with statistical significance set at p ≤ 0.05. To understand the evolution of clinical sequelae over time, a temporal analysis was conducted, and the trends presented in form of a heat map. To assess how long semen and breastmilk samples tested positive, we calculated days persistently positive for each participant. This was defined as the period samples tested positive by PCR after testing positive for Ebola by serum. Duration of SUDV shedding was calculated by determining the difference between the last date the semen or breastmilk sample tested positive and the date the participant was diagnosed with Ebola.

### Ethical considerations

The study protocol was reviewed and approved by the School of Biomedical Sciences Research Ethics Committee at Makerere University (approval # SBS-2022–243) and the Uganda National Council of Science and Technology (approval # HS2618ES). Reliance was provided by Washington State University. Additional administrative approvals were sought from the Ministry of Health Uganda, and the study health facilities. Written informed consents were obtained from all study participants. Those who declined were excluded from the study.

## Results

### Sociodemographic characteristics of Uganda SUDV survivors

The mean age of survivors was 31 (± 14) years while that of controls was 30 (± 13) years (Table 2). There was no significant difference in education level, occupation, or underlying conditions between survivors and controls.

### Comparing systemic clinical symptoms among survivors and controls and controls

**Figure 2:**
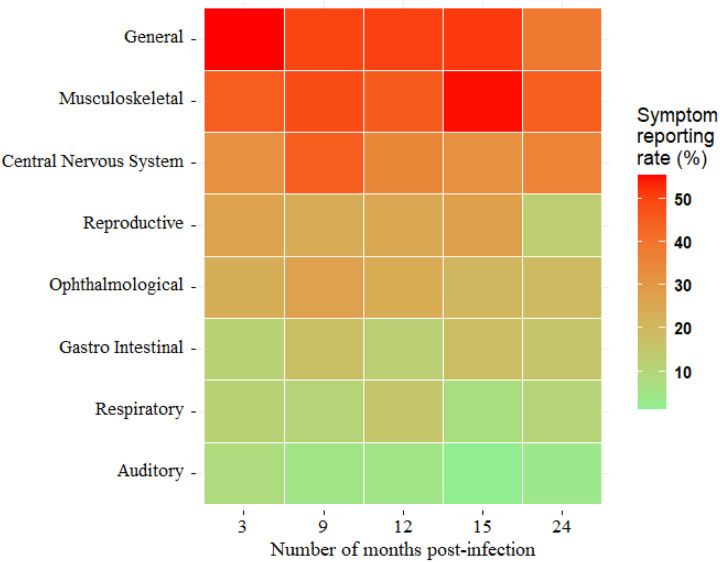
Heat map showing symptom reporting among Sudan ebolavirus (SUDV) survivors over 24 months. This heat map illustrates the proportion of SUDV survivors reporting symptoms across different body systems at 3, 9, 12, 15, and 24 months post-infection. The color gradient represents the symptom reporting rate (%), with red shades indicating higher prevalence (>50%) and green shades representing lower prevalence (<10%).

Overall, 57.5% (50 of 87) of SUDV survivors developed at least one clinical symptom during the follow-up period, when compared to 32.4% of controls (RR = 1.8, p < 0.001). When compared to controls, there was significantly higher frequency of clinical symptoms among survivors for the musculoskeletal, central nervous system (CNS), ophthalmological, reproductive, respiratory, and gastrointestinal systems (P < 0.001, Table 3, Fig. 2). The risk ratios were highest for ophthalmologic and CNS symptoms among SUDV survivors: 5.9 (20% vs 3.4%, P < 0.001) and 5.3 (36.3% vs 6.8%, P < 0.001), respectively, when compared to controls, and lowest in reproductive system symptoms was < 2 (Table 3).

Overall, symptoms related to the musculoskeletal (45%) system were the most frequent symptoms observed among survivors, although these were detected more often in controls as well (Table 3). The number of survivors reporting clinical symptoms was stable over time: 55.2% (48 of 87) at enrolment and 57.5% (46 of 80) at 2 years post-infection. Clinical symptoms associated with general, musculoskeletal, CNS, and ophthalmological systems were sustained at comparable proportions at 3-, 9-, 12-, and 24-months post-infection (Fig. 2).

### Characterization of specific clinical symptoms

As shown in Table 4, the most frequent specific clinical symptoms reported by survivors were memory loss (35.0%), lower back pain (31.3%), hand and feet numbness (25.0%), and headache (21.3%). When compared to controls, the risk ratios of reported memory loss, blurry vision, depression, and sore throat were > 5.5 among survivors, and for reporting joint pains, weakness, eye pain, chest pain, hand and feet numbness, headache, and muscular pain, the risk ratios were between 3 and 5.5 (Table 4).

Of the 46 (57.5%) survivors that reported at least one symptom at two years post-infection time point, 60.9% reported more than three symptoms, 26.1% reported 2–3 symptoms, and 13% reported one symptom. Five of the 46 (10.9%) symptomatic participants reported symptoms that interfered with their daily activities, including hand and feet numbness, lower back pain, reduced libido, confusion, and diarrhoea. Overall, 50% (40/80) of all SUDV survivors reported persistent multiple (> 2) symptom over the 2-year follow-up period.

### Detection of viral RNA in semen and breastmilk for over 6 months

**Figure 3:**
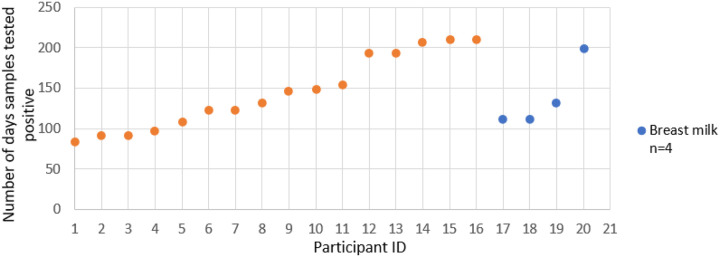
Duration of Sudan ebolavirus (SUDV) RNA detection in semen and breast milk samples. This figure illustrates the number of days that semen (orange, n=16) and breast milk (blue, n=4) samples tested positive for SUDV RNA by PCR. Each dot represents an individual participant.

Of 47 male SUDV survivors aged ≥ 16 years and eligible for semen testing, 33 (70.2%) consented and were tested at enrolment where SUDV RNA was detected in 16 (48.5%) by PCR. Four lactating mothers consented to breastmilk testing and all were PCR positive for SUDV RNA at enrolment. Sixteen (N = 16) viral RNA positive men and the 4 lactating mothers agreed to subsequent testing during monthly psychosocial follow-up visits in accordance with World Health Organization guidelines until two consecutive samples were PCR negative. As shown in Fig. 3, the median duration for semen positivity was 131 days, with the longest duration was 210 days. For breast milk, the median duration of PCR positivity was 149 days with the longest duration of positivity being 199 days.

### Survivor pregnancy outcomes

Four out of five (80%) survivors who became pregnant during the study period delivered babies at term without complications or abnormalities. One (20%) survivor reported spontaneous first-trimester abortion at 12 weeks gestation. All post-partum samples, including amniotic fluid, placental cord blood, breastmilk, baby tears and conjunctival swabs, tested negative for SUDV RNA by PCR.

## Discussion

This study characterized, for the first time, the type and prevalence of clinical symptoms observed during long-term EVD sequelae associated with SUDV, in a case-control approach that enhanced interpretation of findings. At 2-year, statistically significant symptom-specific risk-ratios ranged between 2.9 and 6.8 when compared to controls. Memory loss, lower back pain, hand and feet numbness, and headache were the most prevalent symptoms among survivors. Importantly, symptoms were sustained at comparable levels across the 2-year follow-up period, consistent with studies in EBOV survivors which reported sustained clinical symptoms over the 4 years after infection (5). Over longer periods of time, studies suggest that symptoms appear to resolve for EBOV and BDBV survivors; several studies note higher prevalence of clinical symptoms during convalescent periods when compared to five years later (8, 20, 21) This finding remains to be demonstrated in SUDV survivors. In our study, a substantial proportion of survivors reported debilitating symptoms, such as lower back pain, hand and feet numbness, reduced libido, confusion, and diarrhoea that interfered with wellness and daily living, such as working in the subsistence farming, cooking, and house cleaning.

Some of the high-risk symptoms reported among SUDV survivors were consistent with those reported among EBOV strain survivors, including high prevalence of musculoskeletal and ophthalmological clinical symptoms (6, 22). We found 26.5% and 14.5% of SUDV survivors reporting arthralgia and muscular pain, respectively, compared to 18–87% and 43% among EBOV survivors (22–24). However, the prevalence of some clinical symptoms was different between EBOV and SUDV survivors. For example, while ear, nose, and throat (ENT) symptoms were among the least prevalent (4.8%) in SUDV survivors, a > 5-fold (27%) higher proportion of EBOV survivors reported tinnitus and hearing loss (7, 23). On the other hand, we found a higher prevalence of CNS symptoms including memory loss (35%), hand and feet numbness (25%), and depression (5%), than those reported for EBOV survivors (12% memory loss, 12% hand numbness, 16% feet numbness) (25–27). Whether these differences reflect different viral pathogenesis pathways or differential host immune responses to the virus strains merits further investigations.

Although the pathophysiological mechanisms for long-term clinical sequelae following EVD remain poorly understood, they are speculated to involve either; (i) direct virus pathways by persisting in tissues such as CNS and eye (28); (ii) host immune activation leading to inflammation and tissue damage or autoantibodies-mediated pathophysiology (29), or (iii) both pathways. The ebolavirus ability to evade the immune system and establish latency has been demonstrated (30) and an association between higher viral load during acute EVD and the development of certain sequelae described (31); the later suggesting that the initial severity of the infection may influence likelihood and severity of chronic EVD sequelae.

In our study, there was evidence of persistent virus infection in male and lactating female SUDV survivors, indicated by detection of SUDV RNA in semen and breastmilk for an average of 5 months, and for some men up to 2 years post-infection in semen. Importantly, in two males SUDV RNA was detected in semen eight months after negative results on consecutive samplings, suggesting latency and reactivation of the virus. Similar findings have been reported for EBOV, documenting viral RNA presence for up to 18 months after infection (32, 33). Detection of SUDV RNA in breastmilk signifies a notable risk for vertical transmission from mother to child, while virus in semen confirms the risk of sexual transmission as reported in some studies (33–38). Despite the paucity of documented secondary transmission through these pathways, the potential warrants implementation of informed breastfeeding guidelines for SUDV survivors, and safe sex training for Ebola-discordant couples. Breastfeeding guidelines carefully balance the benefits of breastfeeding with the risks of ebolavirus transmission, optimizing the safety of both survivors and their infants while drawing on the latest research findings to provide optimal evidence-based recommendations (39).

SUDV survivor demographics played a significant role in long-term clinical sequelae, with older survivors reporting joint pain significantly more frequently (χ2=16.87, p = 0.005), while female survivors more commonly reported fatigue (RR = 0.24, p = 0.018), weakness (RR = 0.34, p = 0.015), headache (RR = 0.40, p = 0.029), and depression (RR = 0.14, p = 0.036). These findings mirror those observed in EBOV survivors (5, 40), underscoring distinct age- and gender-related patterns of post-EVD symptomatology. Taken together, these results highlight the need for tailored care strategies that address the unique needs of different demographic groups, as well as further research to re ne management guidelines for EVD survivors.

While we found that 4 of the 5 (80%) female survivors who became pregnant after infection had normal birth outcomes, and that both maternal and foetal postpartum tissues were negative for viral RNA the numbers are too small to conclude minimal risk of vertical transmission. However, studies among a larger cohort of EBOV survivors reported adverse pregnancy outcomes, including high rates of spontaneous abortion and stillbirths, were reported among EBOV survivors(9, 41). These finding suggest continued follow-up studies among pregnant EVD survivors before development of policy guidelines impact of EVD on pregnancy outcomes.

Our study had some limitations. First, the small sample size precluded our ability to identify significant trends in sequelae and viral persistence. Second, the study was observational with self-reporting as the primary source of clinical sequelae data, thus limiting causal inferences. However, follow-up examination of survivors reporting severe symptoms by a trained medical officers strengthened the interpretation of the findings, as did the similarly collected data from matched controls. Data collected from future outbreaks would be helpful, as will continued follow-up of the current cohort to extend over time the characterization of long-term health impacts of SUDV. Our ongoing studies to evaluate the host immune response of the SUDV cohort will likely start to link host immune response to clinical sequelae.

In conclusion, our study demonstrates that SUDV survivors, much like EBOV survivors develop LEDS characterized by persistent multi-systemic debilitating clinical symptoms. Our findings confirm SUDV persistence in survivors with potential for latency and reactivation that may be as source of future human-to-human transmission and outbreaks.

## Figures and Tables

**Table 1:** Symptom information collected during study visits, aggregated by body systems.

Body system	Symptoms
General	Fever, headache, fatigue, weakness, anorexia, weight loss, rash
Musculoskeletal	Joint pain, limb numbness, muscular pain, back pain, joint swelling
Gastro-intestinal	Anorexia, abdominal pain, diarrhoea, vomiting, nausea, difficulty swallowing
Respiratory	Sore throat, difficulty breathing, chest pain, cough
Ophthalmological	Blurry vision, eye pain, watery eyes, vision loss, dry eyes
Reproductive	Reduced libido, testicular pain, erectile dysfunction, menstrual period change
Central nervous system (CNS)	Memory loss, depression, confusion, difficulty in sleeping
Ear, nose, throat (ENT)	Tinnitus, hearing loss

**Table 2:** Sociodemographic characteristics of SUDV survivors and controls at time enrolment

Characteristic	SUDV survivors, N = 87 n (%)	Controls, N = 176 n (%)	p-values
**Sex**			0.7
Male	56 (64.4)	109 (62)	
**Age**			>0.9
Mean (SD)	31 (14)	30 (13)	
Median (IQR)	30 (23, 38)	29 (23, 38)	
**Age Groups**			>0.9
0–9	6 (6.9)	14 (8.0)	
10–19	11 (12.6)	19 (11)	
20–29	30 (34.5)	60 (34)	
30–39	25 (28.7)	50 (28)	
40–49	10 (11.5)	19 (11)	
50+	5 (5.7)	14 (8.0)	
**Level of education**			>0.9
None	45 (51.8)	82 (46.6)	
Primary	16 (18.4)	39 (22.2)	
Secondary	15 (17.2)	33 (18.7)	
Tertiary	11 (12.6)	22 (12.5)	
**Main Occupation**			0.3
Farmer	31 (48.2)	66 (37.5)	
None	17 (19.5)	32 (18.2)	
Healthcare worker	8 (9.2)	3 (1.7)	
Skilled labour*	7 (8.0)	17 (9.7)	
Unskilled labour	5 (5.7)	4 (2.3)	
Other**	19 (21.8)	54 (30.7)	
**Chronic conditions**			NA
Hypertension	6 (6.9)	7 (3.6)	
HIV	5 (5.7)	6 (3.4)	
Diabetes	2 (2.3)	1 (0.5)	
Others	6 (6.9)	2 (1.1)	

* Except healthcare workers

** Businessmen, hairdressers, chefs, carpenters, shop attendants

**Table 3:** Frequency of systemic clinical symptoms among SUDV survivors and controls 12 months after SUDV outbreak.

Body system	Survivors, N = 80 n (%)	Controls, N = 176 n (%)	Risk ratio	95% C. I	p-value
Overall	46 (57.5)	57 (32.4)	1.8	1.3, 2.4	<0.001
Musculoskeletal*	36 (45.0)	25 (14.0)	3.2	2.1,4.9	<0.001
General**	31 (38.8)	34 (19.0)	2.0	1.3,3.0	0.002
Central nervous system***	29 (36.3)	12 (6.8)	5.3	2.9,9.9	<0.001
Ophthalmological ˆ	16 (20.0)	6 (3.4)	5.9	2.4,14.4	<0.001
Reproductive ˆˆ	11 (13.8)	15 (8.5)	1.6	0.8,3.4	0.026
Respiratoryˆˆˆ	8 (10.0)	4 (2.3)	4.4	1.4,14.2	<0.001
Gastro-intestinal ˇ	12 (15.0)	7 (4.0)	3.8	1.5,9.2	0.011
Auditory ˇˇ	3 (3.8)	1 (0.6)	6.6	0.7,62.5	0.092

* muscle pain, joint pain, hand and feet numbness, lower back pain

** Fever, fatigue, general body weakness, headache, weight loss, anorexia

*** stiff neck, confusion, seizures, difficulty sleeping

ˆ eye pain, dry eyes, watery eyes, blurry vision, vision loss

ˆˆ irregular menstrual periods, erectile dysfunction, testicular pain, reduced libido

ˆˆˆ difficulty breathing, chest pain, sore throat depression

ˇ abdominal pain, diarrhoea, nausea, vomiting

ˇˇ hearing loss, buzzing sound in the ears

**Table 4:** Frequency and relative risk of specific clinical symptoms reported by SUDV survivors and controls at 12-months after infection.

Symptoms	Survivors, N = 80 n (%)	Controls, N = 176 n (%)	Risk ratio	95% C. I	P-Values
Memory loss	28 (35.0)	9 (5.1)	6.8	3.4,13.8	<0.001
Lower back Pain	25 (31.3)	19 (10.8)	2.9	1.7,4.9	<0.001
Hand feet Numbness	20 (25.0)	13 (7.4)	3.4	1.8,6.5	<0.001
Headache	17 (21.3)	11 (6.3)	3.4	1.7,6.9	0.001
Weakness	16 (20.0)	7 (4)	5.0	2.2,11.7	<0.001
Joint pain	12 (15.0)	5 (2.8)	5.3	1.9,14.5	0.001
Fatigue	10 (12.5)	15 (8.5)	1.5	0.7,3.1	0.365
Blurry vision	9 (11.3)	3 (1.7)	6.6	1.8,23.7	0.002
Chest pain	7 (8.8)	4 (2.3)	3.9	1.2,12.2	0.039
Eye pain	6 (7.5)	3 (1.7)	4.4	1.1,17.2	0.029
Muscular pain	6 (7.5)	4 (2.3)	3.3	1.0,11.4	0.075
Depression	5 (6.3)	2 (1.1)	5.5	1.1,27.8	0.032
Weight loss	4 (5.0)	5 (2.8)	1.8	0.5,6.4	0.467
Anorexia	3 (3.8)	4 (2.3)	1.7	0.4,7.2	0.681
Sore throat	3 (3.8)	1 (0.6)	6.6	0.7,62.5	0.092

## Data Availability

The datasets generated and/or analyzed during the current study are not publicly available due to participant confidentiality and institutional data protection policies, but are available from the corresponding author on reasonable request.
